# Supplementary material for: How Unawareness of Weight Excess Can Increase Cardiovascular Risk?
Source: J Clin Med. 2022 Aug 23;11(17):4944. doi: 10.3390/jcm11174944 (PMC9456266; doi:10.3390/jcm11174944)

Supplementary materials

Table S1: Clinical characteristics of the study population: Atherosclerotic plaques, Diabetes Mellitus.

Variables	*Total population [N/%]*	*Normal weight (I) [N/%]*	*Unawareness of overweight and obesity (II) [N/%]*	*Awareness of overweight and obesity (III) [N/%]*	*P-value **	*Pairwise comparisons***		
						**IvsII**	**IvsIII**	**IIvsIII**
	N=658	N=256	N=143	N=259				
Atherosclerotic plaques	297 (45.1)	80 (31.3)	72 (49.7)	145 (56.4)	<0.001	sig.	sig,	
	N=640	N=250	N=136	N=254				
DM from the interview	47 (7.3)	5 (2.0)	10 (7.4)	32 (12.6)	0.153	sig.		
Newly diagnosed DM	42 (6.6)	7 (2.8)	10 (7.4)	25 (9.8)	0.271			

The data are shown as N/%; DM - diabetes mellitus; newly diagnosed DM - diabetes diagnosed in the study;
* Pearson's χ^2 test
** tests for 2 proportions with Bonferroni correction
sig. - test result statistically significant after application of Bonferroni correction for multiple comparisons with family-wise error rate of 0.05

Table S2: Family history – obese family members.

Variables	*Total population [N/%]*	*Normal weight (I) [N/%]*	*Unawareness of overweight and obesity (II) [N/%]*	*Awareness of overweight and obesity (III) [N/%]*	*P-value **	*Pairwise comparisons***		
						**IvsII**	**IvsIII**	**IIvsIII**
Obesity in family	176 (30.9)	57 (24.9)	29 (25.4)	90 (39.6)	0.001		sig,	sig.
Mother's obesity	78 (13.7)	20 (8.7)	17 (14.9)	41 (18.1)	0.014			sig.
Father's obesity	60 (10.5)	16 (7.0)	10 (8.8)	34 (15.0)	0.017		sig.	
Sister's obesity	28 (4.9)	6 (2.6)	2 (1.8)	20 (8.8)	0.008		sig.	sig.
Brother's obesity	25 (4.4)	4 (1.7)	2 (1.8)	19 (8.4)	0.001		sig.	sig.

The data are shown as N/%
* Pearson's χ^2 test
** tests for 2 proportions with Bonferroni correction
sig. - test result statistically significant after application of Bonferroni correction for multiple comparisons with family-wise error rate of 0.05

Figure S1: The perception of overweight and obesity among women and men in ROC curves.

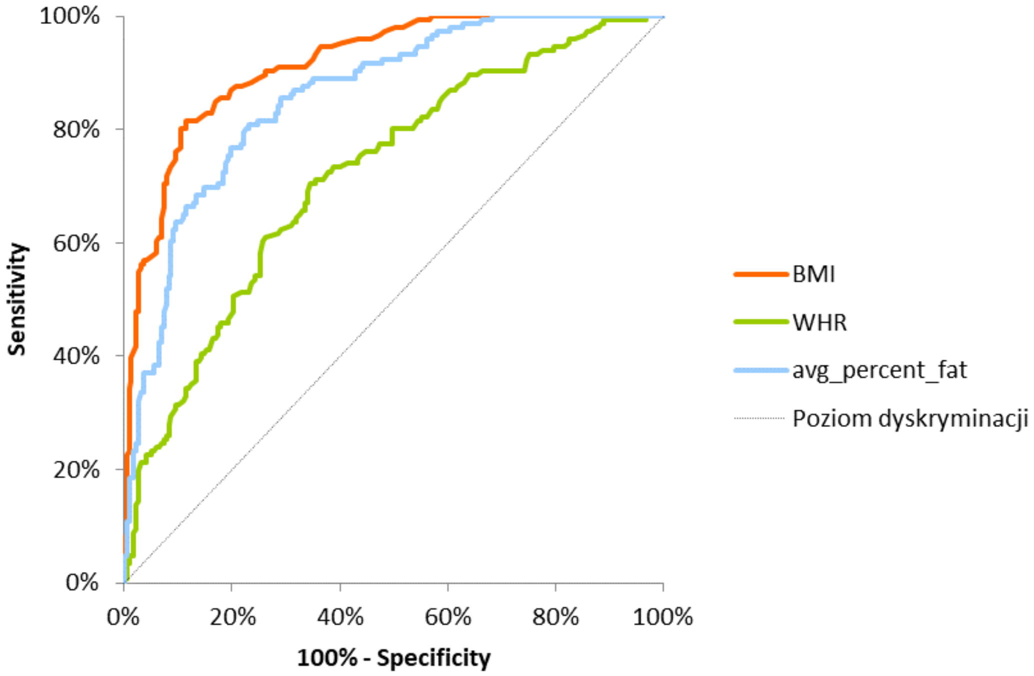

Supplement: Supplementary file 1 [file jcm-11-04944-s001.zip › jcm-1831185-supplementary.pdf]
